# The ties that bind: functional clusters in limb-girdle muscular dystrophy

**DOI:** 10.1186/s13395-020-00240-7

**Published:** 2020-07-29

**Authors:** Elisabeth R. Barton, Christina A. Pacak, Whitney L. Stoppel, Peter B. Kang

**Affiliations:** 1 Center for Exercise Science, Department of Applied Physiology and Kinesiology, University of Florida College of Health and Human Performance, Gainesville, FL USA; 2 Myology Institute, University of Florida, Gainesville, FL USA; 3 Department of Pediatrics, University of Florida College of Medicine, Gainesville, FL USA; 4 Department of Chemical Engineering, University of Florida Herbert Wertheim College of Engineering, Gainesville, FL USA; 5 Division of Pediatric Neurology, Department of Pediatrics, University of Florida College of Medicine, PO Box 100296, Gainesville, FL 32610 USA; 6 Department of Neurology, University of Florida College of Medicine, Gainesville, FL USA; 7 Department of Molecular Genetics and Microbiology, University of Florida College of Medicine, Gainesville, FL USA; 8 Genetics Institute, University of Florida, Gainesville, FL USA

## Abstract

The limb-girdle muscular dystrophies (LGMDs) are a genetically pleiomorphic class of inherited muscle diseases that are known to share phenotypic features. Selected LGMD genetic subtypes have been studied extensively in affected humans and various animal models. In some cases, these investigations have led to human clinical trials of potential disease-modifying therapies, including gene replacement strategies for individual subtypes using adeno-associated virus (AAV) vectors. The cellular localizations of most proteins associated with LGMD have been determined. However, the functions of these proteins are less uniformly characterized, thus limiting our knowledge of potential common disease mechanisms across subtype boundaries. Correspondingly, broad therapeutic strategies that could each target multiple LGMD subtypes remain less developed. We believe that three major “functional clusters” of subcellular activities relevant to LGMD merit further investigation. The best known of these is the glycosylation modifications associated with the dystroglycan complex. The other two, mechanical signaling and mitochondrial dysfunction, have been studied less systematically but are just as promising with respect to the identification of significant mechanistic subgroups of LGMD. A deeper understanding of these disease pathways could yield a new generation of precision therapies that would each be expected to treat a broader range of LGMD patients than a single subtype, thus expanding the scope of the molecular medicines that may be developed for this complex array of muscular dystrophies.

## Key points

- There is a diverse array of genetic subtypes of limb-girdle muscular dystrophy (LGMD).
- The cellular localizations of various proteins associated with LGMD have been characterized, but currently there is little knowledge of unifying disease mechanisms across multiple subtypes.
- We propose that functional clusters of LGMD proteins can illuminate disease mechanisms that are shared across disease subtypes and identify potential therapeutic targets.
- One functional cluster that has been defined better than others is composed of the dystroglycanopathies.
- Two other functional clusters that bear further study are mechanical signaling defects and mitochondrial dysfunction.

## Introduction

In the field of inherited neuromuscular diseases, the primary goal is to identify the underlying genetic cause of the disease, ultimately to understand mechanisms driving pathology, and by doing so, develop effective therapeutic strategies. Nineteen eighty-six was a pivotal year, when the *DMD* gene associated with Duchenne muscular dystrophy (DMD) was discovered [1], followed the next year by the identification of the encoded protein dystrophin [2]. Similarly in the mid-1990s, the *SMN1* gene associated with the most common form of spinal muscular atrophy (SMA) was discovered [3]. These and many other similar discoveries led to international efforts to understand the underlying disease processes and to develop therapeutic strategies targeting the fundamental genetic defects. These endeavors have recently begun to yield remarkable new precision medicines. DMD and SMA are among the most common inherited neuromuscular disorders, yet both are rare diseases; birth prevalence ranges from 15.9 to 19.5 per 100,000 for DMD [4] and 7.8 to 10 per 100,000 for SMA [5, 6]. It is not clear how such precise gene-specific and mutation-specific therapeutic development pipelines can be extended to diseases that are even rarer in an economically sustainable manner. Furthermore, it has become apparent that direct replacement of deficient genes face technical barriers in humans such as immune responses [7] and that, even if successful, such therapies may not necessarily be curative. Specifically, the progression of any neuromuscular disease leads to secondary consequences, including fatty-fibrotic replacement of muscle, and motor unit compression or loss, which may have already occurred by the time the gene therapy is administered [8].

LGMD, a category of muscular dystrophy distinct from DMD, presents two major dilemmas with respect to precision medicine approaches. The first is that even collectively, LGMDs as a whole are rarer than DMD or SMA, with an estimated prevalence of 1.63 to 2.27 per 100,000 [9, 10]; in a large population-based surveillance study, LGMDs composed 9.1% of all muscular dystrophies identified [11, 12]. The second is that unlike these other two diseases, LGMD is composed of an astonishing diversity of genetic etiologies, numbering at least 30 and counting. The numbers have grown so large that they have outgrown the traditional classification system, resulting in a new recently adopted system [13] (Tables 1 and 2). The recessive forms are more common than the dominant ones, with one recent study finding an 84%/16% distribution in a large cohort from Italy [64]. In large, genetically heterogeneous populations, certain subtypes such as LGMD R1 (*CAPN3*), LGMD R2 (*DYSF*), LGMD R3 (*SGCA*), LGMD R4 (*SGCB*), LGMD R5 (*SGCG*), LGMD R6 (*SGCD*), LGMD R9 (*FKRP*), and LGMD R12 (*ANO5*) tend to be relatively common [64, 65], with founder effects in specific regions making it difficult to calculate worldwide prevalence for any particular subtype [12, 66–79]. In contrast, the other subtypes are exceedingly rare except in some genetically isolated populations; many have only been described in certain regions of the world, and there remain a large number of genetically unsolved LGMD cases, both in clinical cohorts [80, 81] and research cohorts [53, 68, 82, 83]. The search for genetic diagnoses in these cases paves the way for potential precision medicine strategies, but the diagnostic odyssey can be quite prolonged for certain individuals, thus delaying definitive genetic counseling and potential eligibility for clinical trials as well as novel therapies that are likely to be approved in coming years.

**Table 1:** Recessive forms of LGMD, listed by the new 2018 classification system [13], with old subtype nomenclature in parentheses

Subtype	Gene	Protein	Cellular localization	Protein function
**LGMD R1 (LGMD2A)**	*CAPN3* [14]	Calpain 3	Myofibril [15]	Cysteine protease
**LGMD R2 (LGMD2B)**	*DYSF* [16, 17]	Dysferlin	Sarcolemma	Membrane resealing [18, 19]
**LGMD R3 (LGMD2D)**	*SGCA* [20]	α-Sarcoglycan	Sarcolemma	Mechanosensor
**LGMD R4 (LGMD2E)**	*SGCB* [21, 22]	β-Sarcoglycan	Sarcolemma	Mechanosensor
**LGMD R5 (LGMD2C)**	*SGCG* [23]	γ-Sarcoglycan	Sarcolemma	Mechanosensor
**LGMD R6 (LGMD2F)**	*SGCD* [24]	δ-Sarcoglycan	Sarcolemma	Mechanosensor
**LGMD R7 (LGMD2G)**	*TCAP* [25]	Telethonin	Sarcomere	Sarcomere assembly and maintenance [26]
**LGMD R8 (LGMD2H)**	*TRIM32* [27]	TRIM32	Myofibril [28]	E3-ubiquitin-ligase
**LGMD R9 (LGMD2I)**	*FKRP* [29]	Fukutin-related protein	Golgi apparatus	Glycosylation
**LGMD R10 (LGMD2J)**	*TTN* [30]	Titin	Sarcomere [31]	Various
**LGMD R11 (LGMD2K)**	*POMT1* [32]	Protein O-mannosyltransferase 1	Endoplasmic reticulum	Glycosylation
**LGMD R12 (LGMD2L)**	*ANO5* [33]	Anoctamin5	Sarcolemma	Membrane resealing
**LGMD R13 (LGMD2M)**	*FKTN* [34, 35]	Fukutin	Golgi apparatus	Glycosylation
**LGMD R14 (LGMD2N)**	*POMT2* [36]	Protein O-mannosyltransferase 2	Endoplasmic reticulum	Glycosylation
**LGMD R15 (LGMD2O)**	*POMGnT1* [37, 38]	Protein O-linked mannose N-acetylglucosaminyltransferase 1	Golgi apparatus	Glycosylation
**LGMD R16 (LGMD2P)**	*DAG1* [39]	Dystroglycan 1	Extracellular matrix	Stabilize sarcomeric cytoskeleton [40]
**LGMD R17 (LGMD2Q)**	*PLEC* [41]	Plectin	Cytosol	Stabilize intermediate filaments [42–44]
**LGMD R18 (LGMD2S)**	*TRAPPC11* [45]	Trafficking protein particle complex 11	Golgi apparatus	Intracellular vesicle trafficking
**LGMD R19 (LGMD2T)**	*GMPPB* [46]	GDP-mannose pyrophosphorylase B	Cytosol	Glycosylation
**LGMD R20 (LGMD2U)**	*ISPD/CRPPA* [47]	CDL-L-ribitol pyrophosphorylase A	Cytosol	Glycosylation
**LGMD R21 (LGMD2Z)**	*POGLUT1* [48]	Protein O-glucosyltransferase 1	Endoplasmic reticulum	Notch signaling
**LGMD R22 (none)**	*COL6A1* *COL6A2* *COL6A3*	Collagen 6α1 Collagen 6α2 Collagen 6α3	Extracellular matrix	Regulation of satellite cell self-renewal and muscle regeneration [49]
**LGMD R23 (none)**	*LAMA2*	Laminin α2	Extracellular matrix	Regulation of autophagy-lysosome pathway [50, 51]
**LGMD R24 (none)**	*POMGNT2*	Protein O-linked mannose N-acetylglucosaminyltransferase 2	Endoplasmic reticulum	Glycosylation
**LGMD R25 (LGMD2X)**	*BVES* [52]	Blood vessel epicardial substance	Sarcolemma	Membrane trafficking [52]
**LGMD R, number pending**	*PYROXD1* [53, 54]	Pyridine nucleotide-disulfide oxidoreductase domain-containing protein 1	Nucleus	Pyridine nucleotide-disulfide reductase [55]

Many of the protein functions listed require further confirmation or are disputed

**Table 2:** Dominant forms of LGMD, listed according to the new 2018 classification system [13], with old subtype nomenclature in parentheses

Subtype	Gene	Protein	Cellular localization	Protein function
**LGMD D1 (LGMD1D)**	*DNAJB6* [56, 57]	DNAJB6	Nucleus (DNAJB6a) [58] Sarcoplasm (DNAJB6b) [58]	Z disc organization [58]
**LGMD D2 (LGMD1F)**	*TNPO3* [59, 60]	Transportin 3	Nuclear membrane	Transports serine/arginine-rich proteins into nucleus [61, 62]
**LGMD D3 (LGMD1G)**	*HNRNPDL* [63]	Heterogeneous nuclear ribonucleoprotein D-like	Nucleus [63]	RNA processing [63]
**LGMD D4 (LGMD1I)**	*CAPN3*	Calpain 3	Myofibril	Cysteine protease
**LGMD D5**	*COL6A1* *COL6A2* *COL6A3*	Collagen 6α1 Collagen 6α2 Collagen 6α3	Extracellular matrix	Regulation of satellite cell self-renewal and muscle regeneration [49]

Many of the protein functions listed require further confirmation or are disputed

## Therapeutic strategies for individual LGMDs

A widening stream of increasingly sophisticated molecular therapies is under development for individual LGMD subtypes. Though none has been approved by the FDA to date, it is becoming increasingly likely that such approvals will occur in the near future. Single gene replacement strategies have been investigated in animal models for some time, primarily using adeno-associated virus (AAV) vectors that contain DNA sequences for individual LGMD genes, such as *CAPN3* [84, 85], *DYSF* [86–89], *FKRP* [90–93], *SGCA* [94–100], *SGCB* [95, 101, 102], and *SGCD* [103–105], and *SGCG* [106, 107]. Such efforts have accelerated and moved into human clinical trials for genes such as SGCG [108], inspired in part by an FDA-approved gene therapy for spinal muscular atrophy [109] and ongoing human studies of microdystrophin gene therapy for DMD [110]. Host immune responses have been a major concern in the implementation of gene therapy for human patients [111]. For various subtypes of LGMD, the residual protein expression may not only explain the milder phenotypes seen in some affected individuals but also has the potential to spare patients from host immune responses to the transgene in the setting of gene therapy approaches [112].

Compensation for and correction of specific mutations has also been studied. Antisense oligonucleotides have been used to rescue specific *DYSF* mutations [113], as well as the most common pathogenic *SGCG* mutation [114, 115], both in mouse models. CRISPR-Cas9 genome editing was used to correct mutations in induced pluripotent stem cells (iPSCs) derived from human patients with *CAPN3* mutations, and those corrected cells were able to treat Capn3 deficiency in a mouse model [116].

There have been some attempts to deliver one LGMD-associated gene using AAV in hopes of rescuing a different LGMD subtype in cases where the two protein products share functional overlap. However, these studies have been largely disappointing to date, including an investigation of *ANO5* delivery into *DYSF* deficient mice, with a rationale that both protein products participate in sarcolemmal membrane resealing [117]. The membrane-resealing pathway has also been targeted via overexpression of MG53, with some promising results in mouse models of *SGCD* [118] and *DYSF* [119] deficiencies. The development of molecular therapies that target more than one LGMD subtype should be feasible. However, it is clear that we do not know enough about common disease mechanisms that cross multiple subtype boundaries, and effective molecular targets are likely to include genes that are not necessarily directly associated with LGMD subtypes.

## Functional clusters in LGMD

Although clinical trials testing a variety of strategies are in progress, there are currently no FDA-approved, disease modifying therapies for any subtype of LGMD [120]. Even as LGMD-targeting gene replacement strategies likely reach approval over the coming years, there will remain LGMD subtypes with low numbers of patients, cohorts of patients with unknown mutations, and patients from a variety of subtypes for whom the potential benefits of a gene or mutation-specific therapy may never exceed the inherent risks associated with these methodologies. Those patients with moderate disease phenotypes regardless of the underlying causative gene mutation would likely fall into a category where there may be interest in testing a pharmacological treatment (that could be halted) but reduced interest in a more permanent experimental strategy. For all of the above-mentioned reasons, the identification of unifying therapeutic targets applicable to multiple subtypes of LGMDs is highly desirable.

**Fig. 1:**
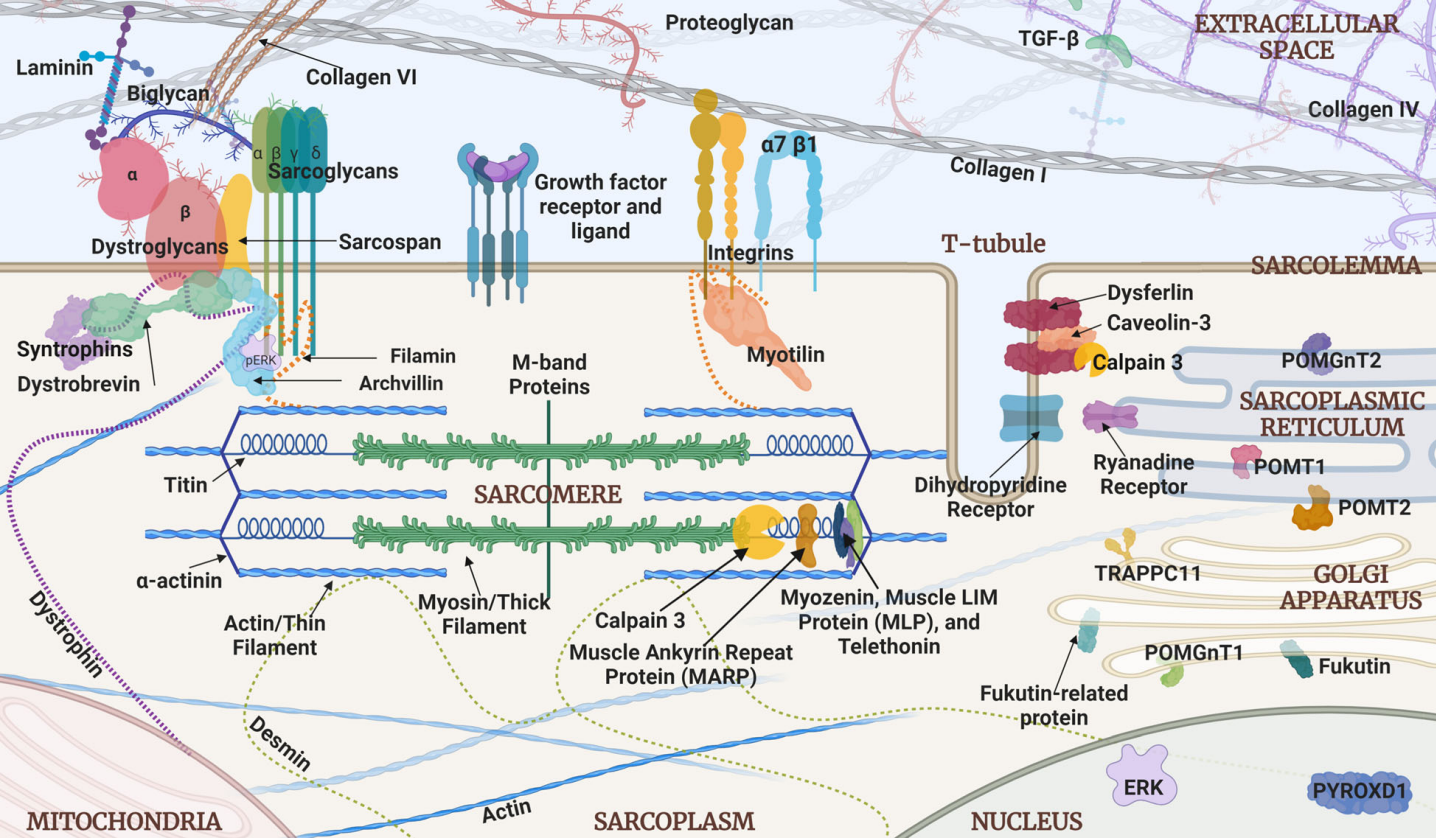
Schematic diagram of proteins associated with LGMD and other muscle diseases such as DMD. When specific proteins are known to interact, they are portrayed as overlapping. The extracellular space occupies the upper portion of the diagram. The double line in the middle represents the sarcolemma. The bottom portion shows the intracellular compartments, including the sarcoplasm, sarcomere, nucleus, and mitochondria. The diverse cellular localizations of proteins associated with both recessive and dominant forms of LGMD highlight the need to organize the proteins into functional clusters that can identify common disease mechanisms and new therapeutic targets. The best known functional cluster to date is the glycosylation pathway that helps create and maintain the dystroglycan complex. The dystroglycanopathy genes include *FKTN*, *FKRP*, *POMT1*, *POMT2*, *POMGnT1*, *POMGNT2*, *ISPD*, and *GMPPB*. The postulated second functional cluster relates to mechanical signaling, which is critical for communications among the contractile apparatus, the surrounding sarcoplasm, the sarcolemma, and the extracellular matrix. The MAPK pathway has been found to be involved in numerous subtypes of LGMD. The sarcoglycan complex in particular is emerging as a key mechanosensor. Other LGMD proteins such as calpain 3 and dysferlin may be additional components of this cluster, or represent independent clusters. The postulated third functional cluster centers around mitochondrial dysfunction, which has been shown to be present in LGMD R1-R6 (LGMD2A-2F), with hints of involvement in newer LGMD genes such as *PYROXD1*

To identify such targets, we should first consider the question: *What binds all of these LGMDs together?* The two core phenotypic features are progressive proximal muscle weakness, along with characteristic signs of muscle fiber destruction on biopsy, referred to as “dystrophic” features. Nuances in clinical presentation have helped to distinguish some of the LGMDs, such as the frequent occurrence of difficulty walking on tiptoes in LGMD R2 (LGMD2B), caused by dysferlin deficiency. However, heterogeneity associated with variable ages of onset and ranges of severity makes it generally difficult to distinguish and diagnose LGMD subtypes based on clinical presentation alone. A change in perspective is in order to aid in understanding disease pathways responsible for clinical features even when the genetic mutation is unknown. Further, given the large number of gene-specific LGMD subtypes, it could very well be that several major disease mechanisms may be shared across the family of diseases. Yet despite careful studies that have collectively determined the cellular localization of most proteins associated with LGMD (Fig. 1), there is limited knowledge of potentially unifying molecular disease mechanisms. We assert that the identification of *functional clusters* of these proteins, grouped by such common mechanisms, will streamline our understanding of the disease processes and identify therapeutic targets relevant to individuals in multiple disease subgroups, including individuals whose pathogenic mutations have not been found. By extension, this approach may serve as a tool to not only find common mechanisms, but may also help to distinguish LGMD subtypes that do not share similar functional patterns, and afford further refinement of potential treatments.

Even though genetic mutations causing LGMD encode a diverse array of proteins (Tables 1 and 2), it is likely that additional functional clusters exist within the LGMD population. A review of the LGMD literature strongly suggests that two potential novel clusters of LGMD focus on defects of mechanical signal transduction and mitochondrial function. As described in the following sections, the mechanical signal transduction cluster may represent a pathway that triggers a maladaptive cascade with each contraction. In contrast, the mitochondrial function cluster may represent a common cellular response to disrupted homeostasis arising through many different triggers, including aberrant mechanical signaling.

## Glycosylation of dystroglycan proteins

The functional cluster that is by far the most fully developed encompasses the dystroglycanopathies. Diseases in this family are bound together by impaired glycosylation of dystroglycans. Genetic mutations that impair this process have been found in a number of genes encoding enzymes, localized primarily at the Golgi apparatus and sarcoplasmic reticulum, that contribute to the normal process of dystroglycan glycosylation (Fig. 1). These genes include *FKRP*, *POMT1*, *FKTN*, *POMT2*, *POMGnT1*, *ISPD*, *POMGNT2*, and *GMPPB* (Table 1). These genes were first associated with severe forms of congenital muscular dystrophy, a related yet distinct category of muscular dystrophy, and then subsequently also associated with milder forms of dystroglycanopathy that met phenotypic criteria for LGMD. The corresponding encoded enzymes fukutin-related protein [29], protein O-mannosyltransferase 1 [121], fukutin [122], protein O-mannosyltransferase 2 [123], protein O-linked mannose N-acetylglucosaminyltransferase 1 [124], CDL-L-ribitol pyrophosphorylase A [125, 126], protein O-linked mannose N-acetylglucosaminyltransferase 2 [127], and GDP-mannose pyrophosphorylase B [46] contribute to the process of O-mannosyl glycosylation of α-dystroglycan before it is transported to the sarcolemma.

Knowledge of the dystroglycanopathies has advanced to the point where experiments have studied the possibility of targeting multiple disease subtypes. One such therapeutic strategy is to overexpress *LARGE*, a dystroglycanopathy gene (currently associated only with a congenital muscular dystrophy phenotype) that induces hyperglycosylation of α-dystroglycan; this approach has augmented glycosylation in mouse models of two other dystroglycanopathies, *FKTN* deficiency and *POMGnT1* deficiency; these latter two genes have been associated with LGMD as well as congenital muscular dystrophy [128]. Another study demonstrated the therapeutic potential of overexpressing *ISPD* in *FKRP* mutant mice [129]. These investigations support the concept that targeting specific components of the glycosylation pathway shared within this functional cluster could provide therapeutic benefit for multiple LGMD subtypes.

## Mechanical signaling defects in LGMD

Skeletal muscle adaptation can occur through changes in active or passive workload converging into final common pathways. During active force generation, multiple signals alter in parallel, including mechanical deformation, phosphorylation patterns, calcium ion fluxes, and local concentration (depletion) of high-energy substrates. Distinct patterns in the MAP kinase family of proteins are evident in healthy tissue in response to active lengthening contractions. In one of the first studies examining this phenomenon, Martineau and Gardiner established a dose response of MAPK phosphorylation with respect to strain and implicated Jnk as the most responsive MAPK to active strain, with ERK1/2 responsive to both active and passive tension, and p38 being relatively insensitive to mechanical deformation [130]. Passive stretch instigates signal transduction within muscle, such as transient induction of the p70S6K pathway [131, 132], but because the energetic cost of active contraction is eliminated, the responses may provide insight into signaling that is more closely aligned with mechanical deformation. Signaling defects are evident in multiple pathways and across many dystrophies. Aberrant signaling is a feature of dystrophic muscle which occurs at rest, during active force generation, and passive movement [131, 133–139], summarized in Table 3.

**Table 3:** MAPK pathway phosphorylation changes associated with mechanical perturbation and neuromuscular diseases in skeletal muscle

Pathway	Disease	Finding	Citations
**Jnk (Thr183/Tyr195)**	Healthy	Jnk activation directly correlated to active tension but not passive tension. P54 is most sensitive	[130]
	Healthy	Resistance exercise stimulates P-JNK	[140]
	DMD (*mdx*)	Elevated P-JNK1 associated with pathology (all refs). JNK1 inhibition by JIP1 attenuates pathology; P54 > p46 in phosphorylation status; Murine diaphragm highest elevation [141]. p46 > p54 in phosphorylation status [142]	[131, 141, 142]
**p38 (Thr180/Tyr182)**	Healthy	Phosphorylated p38 is not sensitive to tension	[130]
	LGMD2C (*Sgcg*^*−/−*^*)*	Stretched myotubes have elevated p-p38	[143]
	FSHD	P38 inhibition reduces DUX4 expression	[144]
	LGMD2B (SJL)	Reduction of P-p38 by paloxamer188 but no comparison to wildtype mice	[145]
	DMD (*mdx*)	Loss of MKP5 improves phenotype	[146]
	LGMD2F (*Sgcd*^*−/−*^); DMD (*mdx*)	Elevated p38 associated with pathology; transgenic ablation of p38a (mapk14) reduces pathology;	[147]
	LGMD2A (C3KO)	Suppressed in sedentary and run conditions	[148]
**ERK1/2 (Thr202/Tyr204)**	Healthy	Erk phosphorylation correlated with both active and passive tension	[130]
	DMD (*mdx*)	Elevated in resting diaphragm, with stretch-dependent enhancement	[149]
	LGMD2C (*Sgcg*^*−/−*^*)*	Stretched myotubes have elevated P-ERK1/2	[143]
	LGMD2C (*Sgcg*^*−/−*^*)*	Resting elevated P-ERK1/2	[133, 134]
	LGMD2C (*Sgcg*^*−/−*^*)*	Sustained levels with contraction but not passive stretch	[131, 134]
	DMD (*mdx*); LGMD2C (*Sgcg*^*−/−*^*)*	Uncoupling of mechano-signaling. Elevated P-ERK1/2 in human biopsies of LGMD2C/2E and DMD	[136]
	LGMD2C	Dusp6 (ERK phosphatase) genetic modifier	[150]
	LGMD2F (*Sgcd*^*−/−*^)	Increased ERK1/2 protects against dystrophy with fast-to-slow fiber type shift; selective ablation of ERK1	[151]

A key contributor to mechanosensing is the sarcoglycan complex. The discovery of α-SG (Sgca) and γ-SG (Sgcg) phosphorylation in response to adhesion of cultured cells led to the proposal that the SG sub-complex regulates mechanosensing [152]. We, and others, have proposed that the SG complex is a critical part of the mechanical signaling machinery and that absence of this complex alters signaling [131, 133, 153, 154]. In a mouse model for LGMD R5 (LGMD2C), ablation of Sgcg causes severe pathology, yet there is little mechanical fragility [155]. However, distinct hyper-signaling through ERK1/2 occurs at rest and with active eccentric contractions with loss of Sgcg [133]. Mechanosignaling may not only originate in the complex but also in modifiers the complex: tyrosine phosphorylation of Sgcg occurs following active and passive strain changes in the muscle [131, 134], and the loss of Sgcg phosphorylation also alters the mechanical response even when the complex is otherwise intact [134]. Indeed, the fact that this residue, as well as others in the intracellular domain, has been associated with severe autosomal recessive muscular dystrophy (https://www.ncbi.nlm.nih.gov/clinvar/?term=Sgcg%5Bgene%5D) point to this region of Sgcg as critical for function. With this in mind, it suggests that proteins involved in modulating the phosphorylation state of the sarcoglycans may also be candidates for LGMD causing mutations or modifiers of the severity of the symptoms.

In contrast to ERK and Jnk, p38 appears relatively insensitive to strain, yet it may still serve as an indication of disease. Activation of alpha and beta isoforms of p38, in particular, are critical regulators of myoblast differentiation [156, 157], hence reduced p38 activity may lead to defects in muscle cell maturation or in regeneration and growth. In addition, p38 is activated during sustained muscle activity, and is one of the upstream triggers for transcription of PPARγ coactivator (PGC)-1, and ultimately mitochondrial biogenesis [158]. Thus, abnormal p38 activity may also underlie maladaptation associated with neuromuscular disease. Both consequences of reduced P-p38 are evident in mice lacking calpain 3 (the protein deficient in LGMD R1 (LGMD2A)). The adaptational response of calpain 3 null mice to exercise training was blunted and associated with diminished P-p38 as well as CAMKII signaling, implicating calpain 3 as an upstream regulator of these signaling pathways [159]. Further, regenerative capacity and recovery from disuse atrophy are also delayed [160, 161]. Even though p38 activity may be depressed with the loss of calpain 3, one cannot extend that disruption to other MAPK proteins, or to aberrant mechanosignaling, and there is no evidence to date to address this possibility.

Not only are there LGMD subtypes with low p38 activity, several studies have demonstrated heightened p38 phosphorylation in mouse and cell models for LGMD R2 (LGMD2B), LGMD R5 (LGMD2C), and LGMD R6 (LGMD2F), as well as facioscapulohumeral muscular dystrophy (FSHD) and DMD [143, 144, 147]. Further suppression of P-p38 ameliorated histopathology in several of these disease models [144, 147], yet dis-inhibition of p38 activity through ablation of the phosphatase, MKP5/Dusp10 also benefited *mdx* muscle [146]. The dichotomy in the direction of aberrant signaling suggests that this pathway must be properly tuned in healthy muscle, but also may serve as a discriminating feature for the specific genetic defect. Namely, defects in the sarcoglycan complex and its associated proteins may share elevated P-p38, whereas defects that are associated with reduced calcium-mediated actions, such as with calpain 3 mutations, may have reduced P-p38. By extension, heightened calcium entry may also exhibit higher P-p38 levels.

How are these LGMD-dependent signaling defects different from aberrant signaling associated with other classes of muscular dystrophies? The loss of dystrophin in DMD also displays aberrant signaling in the MAPK pathways [141, 147, 149]. A common link to a subset of LGMDs is the secondary loss of the sarcoglycan and dystroglycan complexes in DMD, potentially implicating these subcomplexes as the underlying cause of the signaling defects. However, loss of dystrophin is also accompanied by mechanical fragility of the muscle, and this could also contribute to altered signaling. Hence, the separation of mechanical fragility from disrupted mechanical signaling is an important criterion to its identity as a functional cluster.

## Mitochondrial dysfunction in LGMD

An LGMD functional cluster that is based upon mitochondrial function may also help to explain in part the observed variations in age of onset, speed of progression, and overall severity that can vary between patients even when the underlying causative gene is the same [162–164]. This may occur through altered energy production, impaired Ca^2+^ homeostasis, activation of apoptosis, a combination of these, or currently unknown roles for mitochondria in LGMD pathophysiology. Thus far, studies have demonstrated altered mitochondrial function in 6 LGMD subtypes (Table 4), and in one example, variations in mitochondrial-mediated apoptosis due to altered expression of the pro-survival protein BCL2 correlated with disease severity in patients homozygous for the same *SGCG* mutation (LGMD R5, also known as LGMD2C) [53, 147, 163, 165–169]. In sum, these studies strongly suggest that (1) mitochondria impart a key contribution to the pathophysiology of LGMD presentation, and (2) this aspect of dysfunction in LGMD should be more thoroughly investigated across subtypes and individuals to understand which aspects of mitochondrial function are the most viable therapeutic targets.

**Table 4:** Mitochondrial evaluations of LGMDs in literature: listing of mitochondrial assessments and status of these readouts in current LGMD literature

Functional measurement:	Reported analyses in LGMD literature:
**Activation of intrinsic apoptotic pathways**	• Variability in LGMD R5 (LGMD2C) patient severity based upon BCL2 expression levels in skeletal muscle [163] • No activation in aged LGMD R6 (LGMD2F) cardiomyocytes [165]
**Mitochondrial ultrastructure**	• Mitochondrial swelling in LGMD R6 (LGMD2F) cardiomyocytes [165] • Mitochondrial swelling and disorganized structure in LGMD R1 (LGMD2A) patient skeletal muscle [166] • Mitochondrial swelling in LGMD R6 (LGMD2F) [147, 167] • Reduced mitochondrial cristae density in LGMD R3 (LGMD2D) [168]
**Oxygen consumption**	• Reduced in LGMD R3 (LGMD2D) patient and mouse skeletal muscle [168] • Reduced in PYROXD1 knockdown myoblasts [53]
**Mitochondrial membrane potential/permeability transition pore (mPTP) status**	• Decreased potential and open mPTP in LGMD R6 (LGMD2F) [165]
**Electron transport chain (ETC) expression**	• Altered CI and CIV expression in LGMD R2 (LGMD2B) patient muscle [169] • Decreased ETC expression in LGMD R3 (LGMD2D) mouse diaphragm muscle [168] • Decreased CV expression in LGMD R6 (LGMD2F) mouse skeletal muscle and heart [170]
**mtDNA copy numbers**	• Reduced in LGMD R3 (LGMD2D) patient and mouse skeletal muscle [168]
**Mitochondrial biogenesis**	• Defective mitochondrial biogenesis in LGMD R3 (LGMD2D) patient and mouse skeletal muscle [168]
**Mitochondrial Ca**^**2+**^**uptake**	• Ca^2+^ overload in LGMD R6 (LGMD2F) cardiomyocytes [165]

With respect to mitochondrial dysfunction, there is solid evidence from prior work suggesting the presence of mitochondrial dysfunction in several subtypes of LGMD. However, there is no prior research exploring the hypothesis that different forms of mitochondrial dysfunction can help explain the spectrum of phenotypic severity in different subtypes of LGMD. Thus far, no study has systematically examined a thorough panel of mitochondria-related assessments in multiple forms of LGMD. Such studies would enable more optimal selection of patients most likely to benefit from potential mitochondria-targeting treatment strategies based upon their mitochondrial function profile.

## Complementary disease mechanisms and implications for therapy

As noted above, multiple studies indicate that both mechanosignaling and mitochondrial dysfunction contribute to the disease mechanisms of LGMD R1 (LGMD2A), LGMDR5 (LGMD2C), and LGMD R6 (LGMD2F), caused by recessive mutations in *CAPN3*, *SGCG*, and *SGCD*, respectively. It is expected that these two functional clusters, along with glycosylation defects, intersect at various points in their pathways. The interactions among these three have not been explored in depth in prior work, and such explorations would be expected to be quite fruitful with respect to understanding the overall LGMD disease process better. Importantly, the pathways involved in each of these functional clusters can provide parallel evaluation of potential therapeutics, as well as how modulation of one pathway may alter aspects of others.

Genetic and pharmacologic modulation of mechanosignaling and mitochondrial function support the possibility for these functional clusters to become therapeutic targets. The rationale stems from either preventing upstream triggers, such as mechanosignaling, to become pathogenic, or shoring up muscles against the downstream consequences, such as mitochondrial uncoupling. For example, inhibition of MAPK/ERK kinase led to alleviation of cardiac complications in a mouse model of laminA/C (*LMNA*) deficiency [171]. The MAPK pathway has been linked to mechanosignaling in muscle [130]. Metformin has been found to enhance autophagy and provide cardioprotection in a mouse model of δ-sarcoglycan (*SGCD*) deficiency, associated with LGMD R6 (LGMD2F) [172]. The therapeutic effect of metformin in this context appears to arise at least in part from stimulation of mitophagy [173]. Targeting cyclophilin D through Debio 025 also appears to inhibit mitochondria-mediated necrosis in multiple mouse models of muscular dystrophy [167, 174]. These studies hint at the enormous untapped potential of targeting shared disease pathways. However, as these targets are in all cells, the balance between benefit to muscle and detriment to other tissues must be addressed.

## Concluding section

Molecular therapies for selected subtypes of LGMD will almost certainly be approved for clinical use in the next several years, mirroring the revolutionary developments in other neuromuscular diseases. However, the genetic diversity of this disease group and the large number of patients without genetic diagnoses suggest that two parallel tracks of therapeutic development are needed: (1) gene-specific and even mutation-specific precision therapies and (2) broader therapies that target common downstream pathways. Several lines of investigation will enhance the development of the latter: (1) in depth evaluation of existing and new disease models to seek transcriptomic, proteomic, and functional evidence for associations with the postulated functional clusters; (2) exploration of promising model systems such as 3-dimensional scaffolds and Drosophila that are underutilized in the study of LGMD, with examinations of both mechanistic and therapeutic questions; (3) elucidation of the genetic etiologies of individuals affected by LGMD who do not have easily identifiable pathogenic mutations; (4) more precise replication of common and biologically pivotal disease mutations across the spectrum of LGMD subtypes. Further elucidation of disease mechanisms for LGMD will facilitate the development of an array of sophisticated therapeutic approaches that will have a significant beneficial impact on the broadest possible spectrum of patients with this disease.

## Data Availability

Not applicable. All data is compiled from previously published work

## Abbreviations

AAV: Adeno-associated virus
*ANO5*: Anoctamin-5
BCL2: B cell lymphoma 2
*CAPN3*: Calpain 3
DMD: Duchenne muscular dystrophy
*DYSF*: Dysferlin
ERK1/2: Extracellular regulated kinases (MAPK1)
FDA: Food and Drug Administration
*FKRP*: Fukutin-related protein
*FKTN*: Fukutin
iPSCs: Induced pluripotent stem cells
*ISPD/CRPPA*: Isoprenoid synthase domain-containing protein/cytidylyltransferase-like protein-ribitol pyrophosphorylase A
Jnk: c-Jun N-terminal kinase (MAPK8)
*LARGE*: Xylosyl- and glucuronyltransferase 1
LGMD: Limb-girdle muscular dystrophy
MAPK: Mitogen-activated protein kinase
MG53: Mitsugumin-53; tripartite motif-containing 72 (TRIM72)
p38: p38 MAPK (MAPK14)
p70S6K: Ribosomal protein S6 kinase beta-1 (S6K1)
*POMGnT1*: Protein O-linked mannose N-acetylglucosaminyltransferase 1
*POMGNT2*: Protein O-linked mannose N-acetylglucosaminyltransferase 2
*POMT1*: Protein O-mannosyltransferase 1
*POMT2*: Protein O-mannosyltransferase 2
*SGCA* and Sgca: Alpha-sarcoglycan
*SGCB*: Beta-sarcoglycan
*SGCD*: Delta-sarcoglycan
*SGCG* and Sgcg: Gamma-sarcoglycan
SMA: Spinal muscular atrophy
